# Cryptochrome Mediates Light-Dependent Magnetosensitivity of *Drosophila*'s Circadian Clock

**DOI:** 10.1371/journal.pbio.1000086

**Published:** 2009-04-07

**Authors:** Taishi Yoshii, Margaret Ahmad, Charlotte Helfrich-Förster

**Affiliations:** 1 University of Regensburg, Institute of Zoology, Regensburg, Germany; 2 Université Paris VI, PCMP, Paris, France; University of Geneva, Switzerland

## Abstract

Since 1960, magnetic fields have been discussed as Zeitgebers for circadian clocks, but the mechanism by which clocks perceive and process magnetic information has remained unknown. Recently, the radical-pair model involving light-activated photoreceptors as magnetic field sensors has gained considerable support, and the blue-light photoreceptor cryptochrome (CRY) has been proposed as a suitable molecule to mediate such magnetosensitivity. Since CRY is expressed in the circadian clock neurons and acts as a critical photoreceptor of *Drosophila*'s clock, we aimed to test the role of CRY in magnetosensitivity of the circadian clock. In response to light, CRY causes slowing of the clock, ultimately leading to arrhythmic behavior. We expected that in the presence of applied magnetic fields, the impact of CRY on clock rhythmicity should be altered. Furthermore, according to the radical-pair hypothesis this response should be dependent on wavelength and on the field strength applied. We tested the effect of applied static magnetic fields on the circadian clock and found that flies exposed to these fields indeed showed enhanced slowing of clock rhythms. This effect was maximal at 300 μT, and reduced at both higher and lower field strengths. Clock response to magnetic fields was present in blue light, but absent under red-light illumination, which does not activate CRY. Furthermore, *cry^b^* and *cry^OUT^* mutants did not show any response, and flies overexpressing CRY in the clock neurons exhibited an enhanced response to the field. We conclude that *Drosophila*'s circadian clock is sensitive to magnetic fields and that this sensitivity depends on light activation of CRY and on the applied field strength, consistent with the radical pair mechanism. CRY is widespread throughout biological systems and has been suggested as receptor for magnetic compass orientation in migratory birds. The present data establish the circadian clock of *Drosophila* as a model system for CRY-dependent magnetic sensitivity. Furthermore, given that CRY occurs in multiple tissues of *Drosophila,* including those potentially implicated in fly orientation, future studies may yield insights that could be applicable to the magnetic compass of migratory birds and even to potential magnetic field effects in humans.

## Author Summary

Magnetic fields influence endogenous clocks controlling the sleep–wake cycle of animals, but the underyling mechanisms are unclear. Birds that can do magnetic compass orientation also depend on light, and the blue-light photopigment cryptochrome was proposed to act as a navigational magnetosensor. Here we tested the role of cryptochrome as a light-dependent magnetosensor of the clock in the fruit fly Drosophila melanogaster. In wild-type flies we found that constant magnetic fields slowed down the speed of the clock in a dose-dependent manner—but only in the presence of blue light. In mutants lacking functional cryptochrome, the magnetic fields had no significant effects on the endogenous clock, whereas the effects were enhanced after overexpression of cryptochrome. Our data suggest that cryptochrome works as a magnetosensor in the endogenous clock when it is excited by blue light. Our work supports previous data showing that fruit flies need functional cryptochrome to perceive a magnetic field, demonstrating that the interaction of cryptochome and magnetic fields are not just for the birds.

## Abstract 3

The molecular clock of the fruit fly is sensitive to magnetic fields in a manner dependent on blue light and the photopigment cryptochrome.

## Introduction

Endogenous clocks help organisms to adapt to the 24-h cycle of the earth. One of the main characteristics of endogenous clocks is that they continue to oscillate even in the absence of external time cues. Under such conditions, they “free-run” with their endogenous inherited periods that are slightly different from 24 h. Therefore, they are also called circadian clocks. Under natural conditions circadian clocks are synchronized to the 24-h cycle on Earth by different Zeitgebers. The most important Zeitgeber is the light–dark cycle, but temperature, humidity, and the social environment can also serve as Zeitgebers. Additionally, magnetic fields have been discussed as agents that synchronize circadian clocks since 1960, when Brown et al. [1] found that the circadian rhythms of fiddler crabs and other organisms are influenced by small changes in the intensity of Earth's magnetic field. In particular, the electromagnetic field with a frequency of 10 Hz is known to show a prominent 24-h oscillation [2]; thus, the field could serve as geophysical synchronizer of the circadian clock. Although electromagnetic fields are not consciously perceived by humans, Wever [3] demonstrated that the circadian activity rhythm of humans can be entrained by an artificial 10 Hz electromagnetic field, mimicking the natural oscillations on Earth. The activity of mice, house flies, and fruit flies could also be synchronized or phase-shifted by 10 Hz electric fields applied for 12 h daily [4,5]. Cyclically changing intensities of a static magnetic field can also work as weak synchronizers of the circadian clock, as shown by Bliss and Heppner [6] for house sparrows. The latter study was motivated by the idea that birds perform magnetic compass orientation and that this ability depends on a functional clock. Thus, the authors speculated about a link between the magnetosense and the circadian clock.

Despite the compelling effects of (electro)-magnetic fields on the circadian clock, the mechanisms by which the magnetic field is perceived and how it manipulates circadian clocks are unknown. Two main models of magnetic field sensing are currently under discussion: (1) the magnetite model proposing a primary process involving tiny crystals of permanently magnetic material [7], and (2) the radical-pair model suggesting a “chemical compass” based on singlet–triplet transitions in photopigments [8–10].

The radical-pair mechanism, in which the magnetosensor is a photopigment, was first suggested by Schulten and coworkers [8–10]. The first step of the reaction is the absorption of a photon of light energy by the pigment molecule, leading to the transient formation of radical pairs (unpaired electrons) with opposite electron spin states (singlet electrons). The model predicts that these electrons are close enough together that the unpaired electron spin states can undergo transition, at some frequency, from antiparallel to parallel spin states (singlet–triplet intersystem crossing). Such singlet–triplet intersystem crossing is a natural feature of many photochemical reactions that, in fact, may have nothing to do with magnetosensitivity and is the result of the system reaching the most energetically favorable state subsequent to absorption of photon energy. The critical feature of a magnetosensitive radical-pair reaction is that the radicals have a sufficiently long lifetime and are oriented in such a way that singlet–triplet transitions are modified by their alignment in the presence of an applied magnetic field. In such a case, product yield or rate of formation would be different depending on the relative amount of singlet or triplet formation, and these changes in output would provide a means for the organisms to detect the effects of even very weak applied fields. This presumed radical-pair mechanism has indeed been observed experimentally in reactions involving organic compounds [11] and in biological systems such as photosynthetic reaction centers [12,13].

Since the radical-pair mechanism of magnetosensitivity depends on the activation of the relevant photopigment by light, it is wavelength dependent. Magnetosensitivity is maximal at the absorbance maximum of the photopigment, and it depends on the strength of the magnetic field. Ritz et al. [10] calculated the yield of the triplet products in relation to the strength of the magnetic field and the angle of the field with respect to the alignment of the radical pair in space. They found that there is a small window of the magnetic field strength at which the radical-pair mechanism furnishes magnetosensory capacities. In agreement with these calculations, Wiltschko and Wiltschko found that the magnet compass orientation of birds works only at a certain range of magnetic intensities [14].

That magnetoperception is wavelength dependent has been shown not only in birds but also in newts and fruit flies. As photopigments, cryptochromes (CRYs) have been proposed [9,15,16]. CRYs are UV- and blue-absorbing photoreceptors, found in plants and animals, that contain flavin adenine dinucleotide (FAD) as chromophore [17] and have been shown to form radical pairs subsequent to light activation [18]. CRYs are expressed in the eyes of mammals [19] and migratory birds [20,21], where crucial magnetoreceptors have been localized [22,23]. However, CRYs are also expressed in the circadian clock neurons of mice and flies [24–27]. This raises the possibility that magnetic fields can interact directly with the circadian clock.

The fruit fly Drosophila melanogaster is best suited to reveal a putative role of CRYs in the magnetosensitivity of the circadian clock, because there is only one CRY, and because mutants are available that either knock out the binding domain of the FAD (*cry^b^* mutants, [28]) or the entire CRY molecule (*cry^0^* mutants, [29]; *cry^OUT^* mutants, [25]; *cry^Δ^* mutants, [30]). The molecular mechanisms of the circadian clock are largely unraveled. The main players of the clock are the clock genes *period*, *timeless*, *Clock*, and *cycle*, which participate in complex molecular feedback loops to generate the circadian oscillation [31,32]. CRY is known to play an important role in the photoreception pathway to *Drosophila*'s endogenous clock [27]. Light promotes the binding of CRY to TIMELESS (TIM), leading to the degradation of TIM [33,34]. Under light–dark cycles the action of CRY leads to a “reset” of the clock every morning. However, under constant light (LL), TIM-mediated degradation by CRY occurs continuously. Therefore, the amount of TIM is reduced and the clock slows down (i.e., the free-running period of the locomotor rhythm is lengthened). When the constant illumination exceeds a critical intensity (> 10 lx), TIM disappears completely and the clock stops running (the flies become arrhythmic [35]).

On the basis of the background described above we speculated that a magnetic field might influence the circadian clock via CRY under blue light but not red light conditions. To investigate this possibility, we measured the free-running periods of the flies' locomotor rhythms before and during exposure to a magnetic field under constant weak blue light or red light illumination. The blue and red lights were adjusted to such low intensities that in the absence of the magnetic field the flies showed only a slightly longer period than when they were under constant dark (DD) conditions. We found that 40% of the flies further lengthened the free-running periods during the exposure to a magnetic field of 300 μT under blue light illumination. This effect was completely absent under red light conditions. We also demonstrate that the response of fruit flies to the magnetic field is not only wavelength dependent, but also dependent on the strength of the magnetic field, as is implied by the radical-pair model. Furthermore, the magnetic field could not induce any period lengthening in *cry^b^* and *cry^OUT^* mutant flies; but period lengthening was enhanced after overexpressing CRY in the clock neurons. Our results indicate that the magnetic field influences the circadian clock via CRY.

## Results

### Finding the Right Settings

The illumination of the flies was a critical parameter during our experiments, because we intended to activate CRY, but not to such an extent that arrhythmic behavior would occur. We recorded the flies under different intensities of blue light (465–470 nm) and found that 0.18 μW/cm^2^ was a suitable light intensity for the experiments, at which the flies did not become arrhythmic, but showed a significant period lengthening. The mean period at 0.18 μW/cm^2^ blue light was 25.8 ± 0.14 h (mean ± standard error of the mean [SEM], *n* = 25). This period was 1.7 h longer than under DD conditions [36], indicating that CRY was activated by the constant weak blue light and that it provoked a constant weak TIM degradation. Our rationale was that we should see further changes in the free-running period of the flies as soon as they were exposed to the magnetic field affecting the singlet–triplet intersystem crossing of FAD in light-activated CRY.

### Effects of Magnetic Fields on the Free-Running Period

In a first experiment we tested the influence of magnetic fields of different intensities (Figure 1). The radical-pair mechanism predicts that there should be an optimal magnetic strength and that the effects would be smaller under too low or too high magnetic fields [9]. To enable comparison with previous experiments [37] we chose static magnetic fields of 0 μT, 150 μT, 300 μT, and 500 μT; excepting the control of 0 μT these are, respectively, 3, 6, and 10 times stronger than natural magnetic fields. The free-running periods of the flies were determined before and during the application of the constant magnetic fields and the changes in period were calculated (independent of their direction). We found that even flies without exposure to a magnetic field exhibited period changes over the recording time, but that these changes were significantly smaller than those occurring under the influence of a magnetic field of 300 μT (Figures 2A, 2B and 3A). ANOVA revealed that the period changes depended significantly on the strength of the magnetic field (Figure 3A). Most flies lengthened their periods in response to the magnetic field (Figure 2A); but there are also some flies with shortened periods (Figure 2B). To quantify the number of flies with shortened and lengthened periods we defined the following categories (Table 1): Flies exhibiting period changes smaller than 0.5 h (as most wild-type flies did) were defined as flies showing no effects. Flies with shortened or lengthened periods by 0.5 h or more were defined as flies showing period shortening or lengthening, respectively. The third category consisted of flies in which periodogram analysis could not detect any significant period. These were defined as arrhythmic. χ^2^ analysis revealed that the number of flies with lengthened periods was significantly higher when a magnetic field of 300 μT was applied (Table 1).

**Figure 1:**
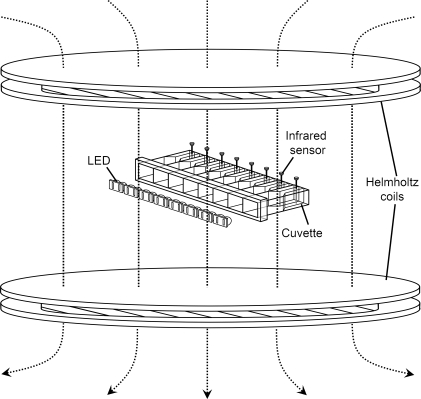
Schematic Representation of the Experimental Apparatus The locomotor recording device was described in detail previously [59,60]. Here eight of 32 spectrophotometer cuvettes are shown as representative “houses” for flies. Two monochromatic LEDs were placed in front of each cuvette, and the flies were illuminated homogenously in the cuvettes. Helmholtz coils were placed above and below the recording device, generating a static magnetic field (dotted light with arrows) perpendicular to the plane of the recording device. The infrared sensors for monitoring the activity of the flies were on the ends of the cuvettes opposite the LED illumination.

**Figure 2:**
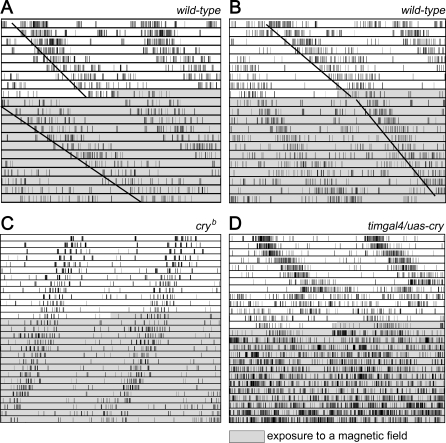
Representative Free-Running Locomotor Rhythms before and during the Exposure to a Magnetic Field Actograms of two wild-type flies (A and B), one *cry^b^* mutant (C) and one CRY-overexpressing fly (*tim-gal4;uas-cry*) (D) are depicted. All flies were exposed to blue light of 0.18 μW/cm^2^ throughout the experiment and to a magnetic field of 300 μT during the second half of the experiment (indicated by gray areas). The free-running periods of 40% of wild-type flies lengthened (A) and 12% shortened (B) during exposure to the magnetic field. These effects were not observed in the majority of *cry^b^* mutant flies (C). More than 60% of CRY-overexpressing flies exhibited arrhythmicity after the magnetic field (D).

**Figure 3:**
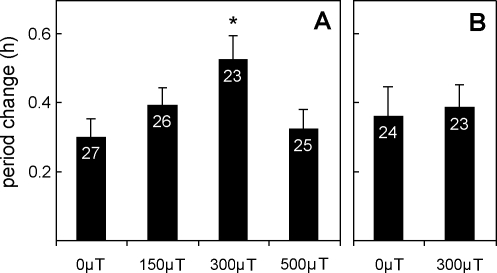
Period Changes (+ SEM) Observed in the Free-Running Rhythm of Wild-Type Flies after Application of Magnetic Fields under Blue Light and Red Light Illumination Under blue light (A), magnetic fields of different strength were applied, and ANOVA revealed that the observed period alterations significantly depended on the strength of the magnetic field (F_3,101_ = 3.0; *p* = 0.03). A post-hoc test revealed that the period change at 300 μT was significantly different from that at 0 μT. Under red light (B), the period alterations produced by a magnetic field of 300 μT were not significantly different from those occurring spontaneously (without a magnetic field). Numbers in the columns indicate the number of included flies. Flies that became arrhythmic are excluded from this analysis (compare Table 1).

**Table 1:**
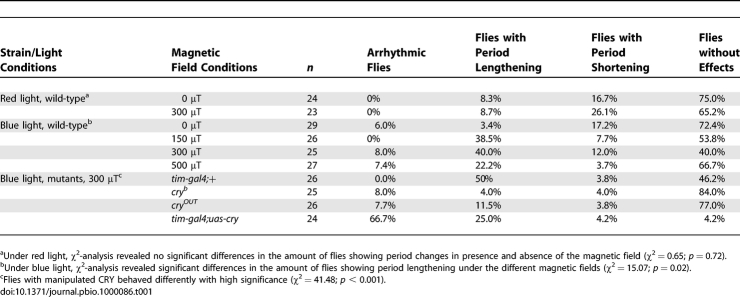
Percentages of flies That Show Alterations in Rhythmic Behaviour after Application of a Magnetic Field

Next we tested whether the effect of the magnetic field depends on the wavelength of the constant light. For that purpose the experiments were performed under a constant red light (625–630 nm) of 0.18 μW/cm^2^, with or without applying a field of 300 μT. We found no difference in period changes between the two groups (Figure 3B; Table 1). This result clearly indicates that the lengthening of the periods by the magnetic field is dependent on the wavelength.

### Cryptochrome Is Involved in Magnetoreception

So far our results are consistent with the idea that magnetoreception involves a radical-pair mechanism occurring in a photopigment. In the next step we wanted to know whether CRY might be the relevant photopigment, so we tested *cry^b^* mutants in which FAD binding is impaired [38] and *cry^OUT^* mutants that lack CRY completely [25]. As expected, both mutants show short periods under blue light of 0.18 μW/cm^2^ (Figure 2C), because TIM is not degraded due to the mutation in the CRY protein (e.g., [34]). The free-running periods of *cry^b^* and *cry^OUT^* mutants were 23.4 ± 0.05 h (*n* = 25) and 23.7 ± 0.04 h (*n* = 26), respectively. After exposure to the 300 μT magnetic field, 88.0% of *cry^b^* flies and 76.9% of *cry^OUT^* flies did not show any period changes (Figure 2C; Table 1), and when changes occurred these were significantly smaller than those in wild-type flies (Figure 4). This indicates that CRY is involved in the magnetically sensitive response of the fly.

**Figure 4:**
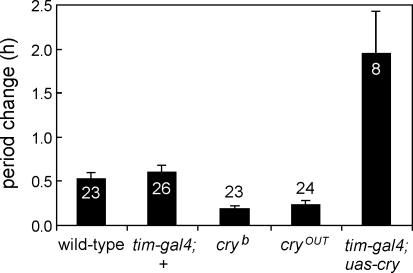
Period Changes (+ SEM) Observed in the Free-Running Rhythm of Wild-Type and Control Flies and Fly Strains with Manipulated CRY after Application of a Magnetic Field (300 μT) and Blue Light In *cry^b^* mutants CRY cannot bind its chromophore flavin, *cry^OUT^* mutants are devoid of CRY, and *tim-gal4;uas-cry* flies have CRY overexpressed in all clock neurons. ANOVA revealed a significant influence of the strain on the period change (F_3,76_ = 29.88; *p* < 0.001). A consecutive post-hoc test revealed that *cry^b^* and *cry^OUT^* mutants exhibited significantly less period change than CRY-overexpressing flies, which showed significant larger period changes than all other strains. Numbers in or above the columns indicate the number of included flies. Since most *tim-gal4;uas-cry* flies became arrhythmic in the magnetic field (Table 1) only eight flies could be included in the analysis.

Next we wanted to test whether we could not only diminish the responses to the magnetic field by knocking out CRY, but also increase the responses by overexpressing CRY in the clock neurons. This test could be done by expressing CRY under the control of the strong clock-gene promoter of the *timeless* gene (in *timgal4/uas-cry* flies). The circadian clock of such flies shows increased light sensitivity [38], and accordingly we found that CRY-overexpressing flies showed significantly longer free-running periods than wild-type flies under blue light. This result indicates that the higher amount of light-activated CRY degrades TIM to a greater extent. The average period of the 24 tested CRY-overexpressing flies was 26.9 ± 0.33 h, and one fly showed arrhythmicity. After exposure to the magnetic field, most CRY-overexpressing flies became arrhythmic (Figure 2D; Table 1) and the remaining ones showed large period changes (Figure 4). All flies except one lengthened their free-running periods (Table 1). These results show that the degree of responsiveness to the magnetic field is directly linked to the levels of CRY present in the fly, and that the site of action of the magnetic field effect can be directly localized to the clock neurons, in which CRY is active in setting the circadian clock.

## Discussion

We show here that *Drosophila*'s clock is magnetosensitive, and that this sensitivity depends on CRY. When the first studies were performed that showed magnetosensitivity of the circadian clock, CRY had not been detected yet and the mechanism of how the clock is influenced by magnetic fields was completely elusive. Now, we know that CRY is expressed in the clock neurons of *Drosophila* [25,30], that CRY absorbs UV and blue-green light [39], and that CRY consecutively binds to TIM and induces its degradation [34]. Under constant conditions this process leads to period lengthening or arrhythmicity [35]. The magnetic field effect that we observed in our study led to further lengthening of the period, in the same way as an increased intensity of blue light would lead to enhanced CRY function. The effect of a magnetic field was particularly pronounced in CRY-overexpressing flies, in which small differences in CRY function should be significantly amplified. Additionally, we could show that this reaction depends on the strength of the magnetic field and on the wavelength of the light. Red light that is outside of the absorption and action spectra of CRY [39–42] did not provoke significant responses. Consistent with the involvement of cryptochrome, *cry^b^* and *cry^OUT^* mutants respond neither to blue-green light nor to the magnetic field.

Our results strongly support the radical-pair model suggesting light-activated flavin-based photoreceptors as sensors for magnetic fields. In several animals magnetoreception has been shown to depend preferentially on blue light [43–47]. Ritz et al. [10] stated first that CRYs are suitable molecules for such photoreceptor-based magnetoreception. CRYs are blue-green photoreceptors in plants and flies [17,28,48,49] and have been shown to form radical pairs upon photoexcitation [18] as is true for the closely related DNA photolyases [50]. Other classes of photoreceptors, such as phototropins [51] and chlorophylls [52] found in plants, also undergo radical-pair reactions. However, rhodopsins, which are the major photosensory pigments in flies and other animals, are not able to form radical pairs because photoexcitation leads to *cis*–*trans* isomerization of retinal rather than an electron transfer [53]. Thus, CRYs are the only currently known class of molecules found in animals that are likely to fulfill the physical and chemical characteristics that are required for functioning as the primary magnetic sensor.

The first evidence that CRYs may work as magnetosensors comes from the plant Arabidopsis thaliana, where it was shown that CRYs mediate magnetic field-dependent hypocotyl growth inhibition under blue light [37]. In these experiments, the effect of the magnetic field was to increase the plant's response to perceived blue light by enhancing cryptochrome activity, similarly to the presently observed magnetic field effect in flies. The second evidence comes from the fly D. melanogaster (that paper was published during the preparation of the present manuscript [54]). Gegear et al. [54] showed clearly that fruit flies need functional CRY to perceive a magnetic field that was paired with a sugar reward during a classical learning experiment. Although this study did not analyze the effect of magnetic field strength on CRY activity, these data are highly consistent with our present observations that the magnetic field intensifies the effects of blue light on the clock. The radical-pair mechanism predicts that the rate or yield of product formation from such a reaction will be altered by an applied magnetic field. Therefore, the pairing of blue light and the magnetic field may lead to the perception of more intense (brighter) light as compared to blue light of the same intensity presented alone. Consequently, hypocotyl growth was inhibited to a higher degree in *Arabidopsis* [37], and flies learned to correlate a more intense light signal under magnetic field presence with the sucrose award [54]. In our experiments the circadian clock lengthened its period or became arrhythmic as if it experienced blue light of a higher intensity. All three phenomena clearly do not depend on directional effects of the magnetic field.

That flies can also perform magnetic compass orientation was shown first by Wehner and Labhart [55] and further elaborated by Phillips and Sayeed [43] and Dommer et al. [47]. The latter two studies showed that *Drosophila* adult males and larvae are capable of learning the alignment of important environmental gradients (i.e., light, food, and humidity) with respect to the geomagnetic field [43,47]. These responses were maximal under UV light, but the involvement of CRY was not tested.

The ability of animals to detect geomagnetic fields has substantial biological importance, because it is used by invertebrate and vertebrate species for orientation and navigation [56]. Almost certainly CRY also plays a crucial role in this process (see [10,16,20,21,57] for discussion). Fruit flies may be well suited to further unraveling the involvement of CRY in the mechanisms of magnetic compass orientation. Since CRY is also present outside the clock neurons in tissues such as the compound eye and the ellipsoid body [25], in which it might show a directional alignment, it will be most rewarding to test the roles of these structures in magnetic compass orientation.

In conclusion, we provide the first evidence of a mechanistic link between CRY and magnetic sensitivity in the circadian clock of *Drosophila*. Our data provide powerful support for the radical-pair hypothesis, because they show the predicted relationship between strength of the applied field and the reaction (here period of the clock). Furthermore, the fact that the magnetic field leads to enhanced photosensitivity of CRY in both plants and flies argues for a similar mechanism of magnetoperception in all organisms that contain CRYs, including humans. Finally, given the recent observations that sun compass orientation in the monarch butterfly is dependent on the circadian clock and on CRY [57], this effect of magnetic field on the circadian clock may in fact play a role in fly orientation.

## Materials and Methods

### Fly strains.

*CantonS* was used as wild-type strain and compared with the *cry* mutants *cry^b^* [28] and *cry^OUT^* [25]. For CRY overexpression in all clock neurons, *w;tim(UAS)gal4* flies [58] and *w;UAS-cry* flies [38] were crossed. All flies were reared under 12:12 light-dark cycles on *Drosophila* medium (0.8% agar, 2.2% sugar beet syrup, 8.0% malt extract, 1.8% yeast, 1.0% soy flour, 8.0% corn flour, and 0.3% hydroxybenzoic acid) at either 20 °C or 25 °C. Only adult male flies at an age of 3–6 d were used for the experiments.

### Recording the locomotor activity of flies.

Locomotor activity of individual male flies was recorded automatically with infrared light beams at constant 20 °C as described previously [59,60]. Briefly, the flies were confined to photometer cuvettes that were placed with one end in an infrared light beam. The number of light beam crosses by a walking fly was monitored during consecutive 1-min intervals. Monochromatic LEDs (Lumitronix LED-Technik, Jungingen, Germany) emitting blue (465–470 nm) and red (625–630 nm) light were used as light sources to illuminate the flies. Light intensity of both LEDs was adjusted to 0.18 μW/cm^2^. Under constant blue light of 0.18 μW/cm^2^, flies displayed slightly longer free-running periods than those in DD, but did not become arrhythmic. To expose the flies to magnetic fields, Helmholtz coils of sufficient diameter (20 cm) to cover the locomotor recording device were placed above and below the recording device, generating a perpendicular static magnetic field (DC mode of current) (Figure 1). The magnetic field intensity was controlled by changing the voltage of the power supply that connects to the coils and was placed outside the recording device. The magnetic intensities were measured by a gaussmeter (475 DSP Gaussmeter, Lake Shore Cryotronics, Ohio, United States). To generate magnetic fields of 150, 300, and 500 μT, 6, 12, and 20 volts were given to the coils, respectively. For comparison, the natural magnetic field of the Earth ranges between 30 μT (equator) and 60 μT (poles). We used static magnetic fields of 150–500 μT instead of oscillating magnetic fields of natural strength to compare our results directly with former results on A. thaliana [37]. The free-running rhythms were first recorded for 10–15 d without a magnetic field, and then fields of four different intensities (0, 150, 300, 500 μT) were applied. All experiments were performed in the same recording device that was positioned in a climate chamber. The climate chamber was surrounded by a metal case shielding the flies from the local geomagnetic field. In the absence of the applied artificial magnetic fields the Gaussmeter could not detect any magnetic field strength in the chamber containing the flies. Furthermore, the climate chamber was tightly temperature controlled. Ventilators close to the Helmholtz coils immediately blew away any heated air. A maximum of 0.5 °C temperature fluctuations were measured close to the Helmholtz coils. Such small temperature changes are known not to influence the circadian clock.

### Data analysis.

The raw data were displayed as actograms using the program El Temps (Antoni Diez-Noguera, Barcelona, 1999; http://www.el-temps.com). To evaluate the periods of the free-running rhythms, data for 10 d before and during the exposure to the magnetic field was chosen and the periods were calculated by the χ^2^ periodogram analysis [61]. This is an objective method making a double-blind experimental approach needless. In the control flies that did not experience any magnetic field, exactly the same subjective days of the flies were used for period determination. If a peak above the 0.05 confidence level appeared in the periodogram, the period was designated as statistically significant.

### Statistics.

A one-way ANOVA was used to test for significant influences of the magnetic fields or of CRY on the amount of period changes. A subsequent post hoc test with Bonferroni's adjustment was applied for pairwise comparisons (Systat 11; SPSS, Chicago, Illinois, United States). χ^2^ analysis was used to test whether the number of arrhythmic flies or the number of flies showing period lengthening increased significantly upon exposure to the magnetic field. Values were regarded as significantly different at *p* < 0.05.

## Abbreviations

CRY: cryptochrome
DD: constant darkness
FAD: flavin adenine dinucleotide
LED: light-emitting diode
LL: constant light
SEM: standard error of the mean
TIM: Timeless
UV: ultraviolet
